# Crystallization and preliminary X-ray crystallographic analysis of YfcM: an important factor for EF-P hydroxylation

**DOI:** 10.1107/S2053230X14015726

**Published:** 2014-08-27

**Authors:** Kan Kobayashi, Takehiro Suzuki, Naoshi Dohmae, Ryuichiro Ishitani, Osamu Nureki

**Affiliations:** a Department of Biological Sciences, Graduate School of Science, The University of Tokyo, 2-11-16 Yayoi, Bunkyo-ku, Tokyo 113-0032, Japan; b Global Research Cluster, RIKEN, 2-1 Hirosawa, Wako, Saitama 351-0198, Japan

**Keywords:** elongation factor P, *in situ* proteolysis crystallization

## Abstract

Elongation factor P (EF-P) plays an essential role in the translation of polyproline-containing proteins in bacteria. It becomes functional by the post-translational modification of its highly conserved lysine residue. It is first β-lysylated by PoxA and then hydroxylated by YfcM. In this work, the YfcM protein from *Escherichia coli* was overexpressed, purified and crystallized. The crystal of YfcM was obtained by the *in situ* proteolysis crystallization method and diffracted X-rays to 1.45 Å resolution. It belonged to space group *C*2, with unit-cell parameters *a* = 124.4, *b* = 37.0, *c* = 37.6 Å, β = 101.2°. The calculated Matthews coefficient (*V*_M_) of the crystal was 1.91 Å^3^ Da^−1^, indicating that one YfcM molecule is present in the asymmetric unit with a solvent content of 35.7%.

## Abstract 2

*E. coli* YfcM was expressed, purified and crystallized. Crystals of YfcM were obtained by the *in situ* proteolysis crystallization method. Using these crystals, an X-ray diffraction data set was collected at 1.45 Å resolution.

## 1. Introduction   

Elongation factor P (EF-P) is a bacterial homologue of eIF5A (eukaryotic initiation factor 5A) and its shape is similar to that of tRNA (Hanawa-Suetsugu *et al.*, 2004 ▶). It binds to ribosomes and stimulates peptide-bond formation (Glick & Ganoza, 1975 ▶; Glick *et al.*, 1979 ▶). The crystal structure of the ribosome in complex with EF-P, initiator tRNA and mRNA showed that EF-P interacts with the ribosome between the P and E sites, where it forms several interactions with the initiator tRNA in the P site (Blaha *et al.*, 2009 ▶). EF-P was considered to facilitate the formation of the first peptide bond during translation initiation by positioning the initiator tRNA properly (Blaha *et al.*, 2009 ▶). 

However, recent studies have revealed a new function of EF-P: the translation of polyproline-containing proteins during translation elongation (Ude *et al.*, 2013 ▶; Doerfel *et al.*, 2013 ▶). In this reaction, the post-translational modification of the highly conserved Lys34 of EF-P is crucial. Firstly, the Lys34 is β-lysylated by PoxA, which is a lysyl-tRNA synthetase paralogue (Yanagisawa *et al.*, 2010 ▶; Roy *et al.*, 2011 ▶). The Lys34 of EF-P structurally corresponds to the aminoacylation site of tRNA, which enables PoxA to attach the (*R*)-β-lysine to Lys34. The β-lysylation strongly stimulates the activity of EF-P (Park *et al.*, 2012 ▶; Doerfel *et al.*, 2013 ▶). Lys34 of EF-P is then further hydroxylated at C4(γ) or C5(δ) by YfcM (Peil *et al.*, 2012 ▶). The functional role of the hydroxylation of EF-P by YfcM is unclear. YfcM lacks amino-acid sequence similarity to any previously reported hydroxylation enzyme. To determine the crystal structure of YfcM and the mechanism of EF-P hydroxylation by YfcM, YfcM from *Escherichia coli* was overexpressed in *E. coli* and purified. Using the *in situ* proteolysis crystallization method (Dong *et al.*, 2007 ▶), crystals of YfcM were obtained which diffracted X-rays to 1.45 Å resolution.

## 2. Materials and methods   

### 2.1. Macromolecule production   

The gene encoding full-length YfcM was amplified by PCR from *E. coli* genomic DNA and cloned into the pET-26b vector (Novagen). The plasmid was introduced into *E. coli* strain Rosetta2(DE3) (Novagen) and YfcM was expressed as a C-terminally His-tagged protein. The cells were grown at 310 K in 2.5 l LB medium to an OD_600_ of 0.5–0.6. Protein expression was induced with 0.5 m*M* isopropyl β-d-1-thiogalactopyranoside at 293 K overnight. The cells were suspended in 100 ml buffer *A* (20 m*M* Tris–HCl pH 8.0, 200 m*M* NaCl, 20 m*M* imidazole, 1 m*M* β-mercaptoethanol) containing 0.1 m*M* phenylmethylsulfonyl fluoride and lysed by sonication. After centrifugation, the supernatant was applied onto a 5 ml Ni–NTA Superflow column (Qiagen). The column was washed with 75 ml buffer *A* and YfcM was eluted with 30 ml buffer *A* containing 300 m*M* imidazole. The eluted fraction was mixed with 20 ml buffer *B* (20 m*M* Tris–HCl pH 8.0, 3.0 *M* ammonium sulfate, 1 m*M* β-mercaptoethanol) and centrifuged. The supernatant was applied onto a 6 ml Resource PHE column (GE Healthcare), equilibrated with buffer *C* (20 m*M* Tris–HCl pH 8.0, 1.2 *M* ammonium sulfate, 1 m*M* β-mercaptoethanol). YfcM was eluted with a linear gradient of 1.2 to 0 *M* ammonium sulfate. The fractions containing YfcM were collected and concentrated by ultrafiltration to 5 ml. YfcM was further purified by chromatography on a Hiload 16/60 Superdex 75 column (GE Healthcare) equilibrated with buffer *D* (20 m*M* Tris–HCl pH 8.0, 200 m*M* NaCl, 1 m*M* β-mercaptoethanol). Macromolecule-production information is summarized in Table 1 ▶.

### 2.2. Crystallization   

YfcM was concentrated by ultrafiltration to 15.7 mg ml^−1^. The 6.3 mg ml^−1^ YfcM solution was used for crystallization screening. Crystallization of YfcM was performed by the sitting-drop vapour-diffusion method at 293 K. A Mosquito crystallization robot (TTP Labtech) was used to mix 100 nl protein solution with 100 nl reservoir solution in a 96-well VIOLAMO crystallization plate (AS ONE). The following crystallization screening kits were used: Crystal Screen, Crystal Screen 2, Index (Hampton Research), Wizard I, Wizard II (Emerald Bio), MemGold (Molecular Dimensions), The PACT Suite and The JCSG+ Suite (Qiagen). However, even small crystals were not generated under these conditions. We then used the *in situ* proteolysis crystallization method (Dong *et al.*, 2007 ▶). The 7.9 mg ml^−1^ YfcM solution was incubated with a small amount of trypsin [1:100(*w*:*w*)] and used for crystallization screening in the same manner as above. Under these conditions, small crystals were generated under several conditions containing PEG in 1 d. Initial crystallization conditions were optimized by varying the concentrations of salt and PEG. Furthermore, the size and shape of the crystals were improved by the hanging-drop vapour-diffusion method. Crystals were grown in 4 µl drops prepared by mixing 2 µl protein solution and 2 µl reservoir solution. Crystallization information is given in Table 2 ▶.

### 2.3. Data collection and processing   

The crystal of YfcM was soaked in a cryoprotectant solution consisting of 27% PEG 3350, 210 m*M* ammonium sulfate, 120 m*M* bis-tris pH 5.5, 20% ethylene glycol. The crystals were then flash-cooled at 100 K. X-ray diffraction data were collected on beamline BL32XU at SPring-8, Hyogo, Japan using an MX225HE detector. A data set was collected using a 7 × 15 µm beam at a wavelength of 1 Å in the presence of a 1.5 mm aluminium attenuator. The total oscillation angle was 360° (1° per frame) and the exposure time was 1 s per frame. The diffraction data set was subsequently processed with *HKL*-2000 (HKL Research, Otwinowski & Minor, 1997 ▶). Data-collection statistics are given in Table 3 ▶.

## 3. Results and discussion   

**Figure 1:**
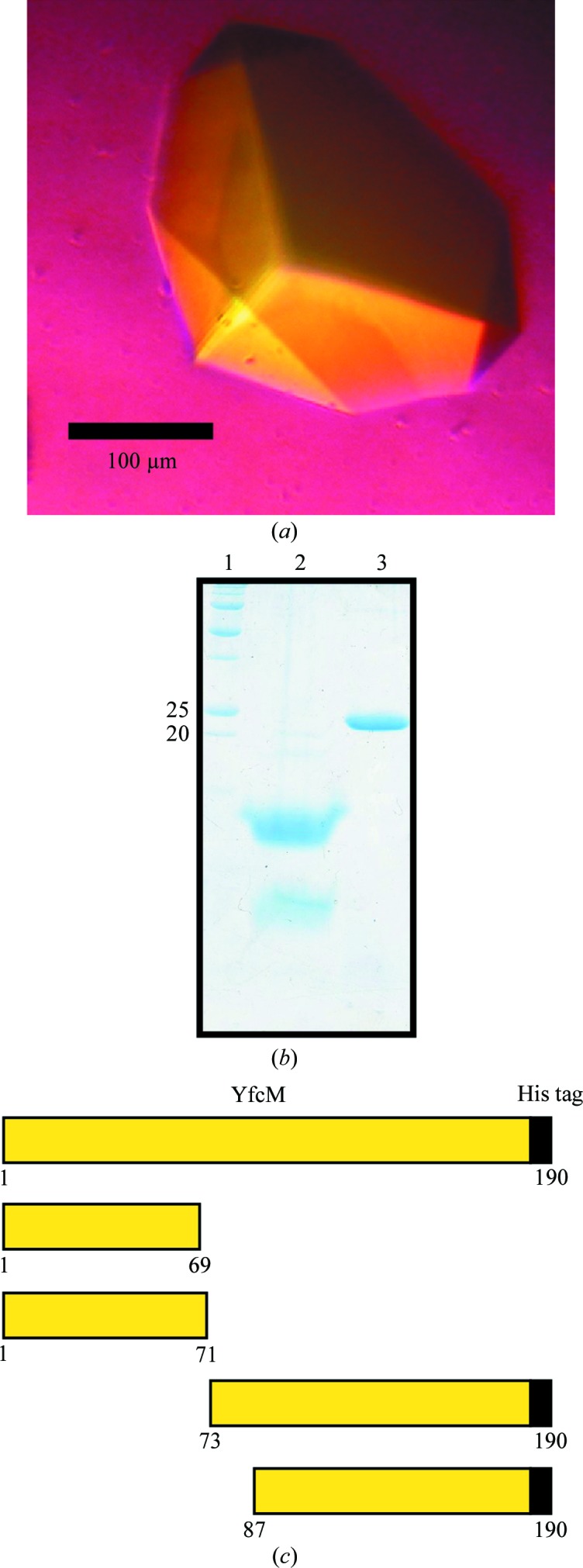
The crystal of YfcM and its analysis. (*a*) The native crystal obtained under the optimized condition. (*b*) Analysis of native crystals by SDS–PAGE. Lane 1, molecular-weight marker (labelled in kDa). Lane 2, crystal. Lane 3, purified YfcM. (*c*) Schematic diagrams of the full-length YfcM crystallization construct (top) and the four YfcM fragments detected in the crystals.

As a result of the crystallization screening, crystals of YfcM only appeared in the presence of trypsin in 1 d. By optimization of the crystallization conditions, a crystal with dimensions of 200 × 200 × 100 µm was obtained using a reservoir condition consisting of 26% PEG 3350, 180 m*M* ammonium sulfate, 100 m*M* bis-tris pH 5.5 (Fig. 1 ▶ *a*).

We tried to determine how trypsin enabled the crystallization of YfcM. We harvested crystals of YfcM, washed them with the reservoir solution and dissolved them in water. The solution was analyzed by SDS–PAGE, N-terminal amino-acid sequencing and MALDI–TOF mass spectrometry. The SDS–PAGE results showed that the crystals consisted of two trypsin-digested YfcM fragments (Fig. 1 ▶ *b*). We next determined the trypsin-cleavage sites in the crystallized YfcM. Our N-terminal sequencing analysis detected YfcM fragments starting from Met1, Glu73 and Asp87. Furthermore, our MALDI–TOF mass-spectrometry analysis detected four YfcM fragments: Met1–Lys69, Met1–Arg71, Glu73–C-terminus and Asp87–C-terminus (Fig. 1 ▶ *c*). These results are consistent with our SDS–PAGE analysis of the crystals showing two bands, with the upper band corresponding to the fragments Glu73–C-terminus and Asp87–C-terminus and the lower band to the fragments Met1–Lys69 and Met1–Arg71. Therefore, partial digestion of YfcM by trypsin enabled crystallization. It is possible that the digested region of YfcM is flexible and prevents crystallization.

**Figure 2:**
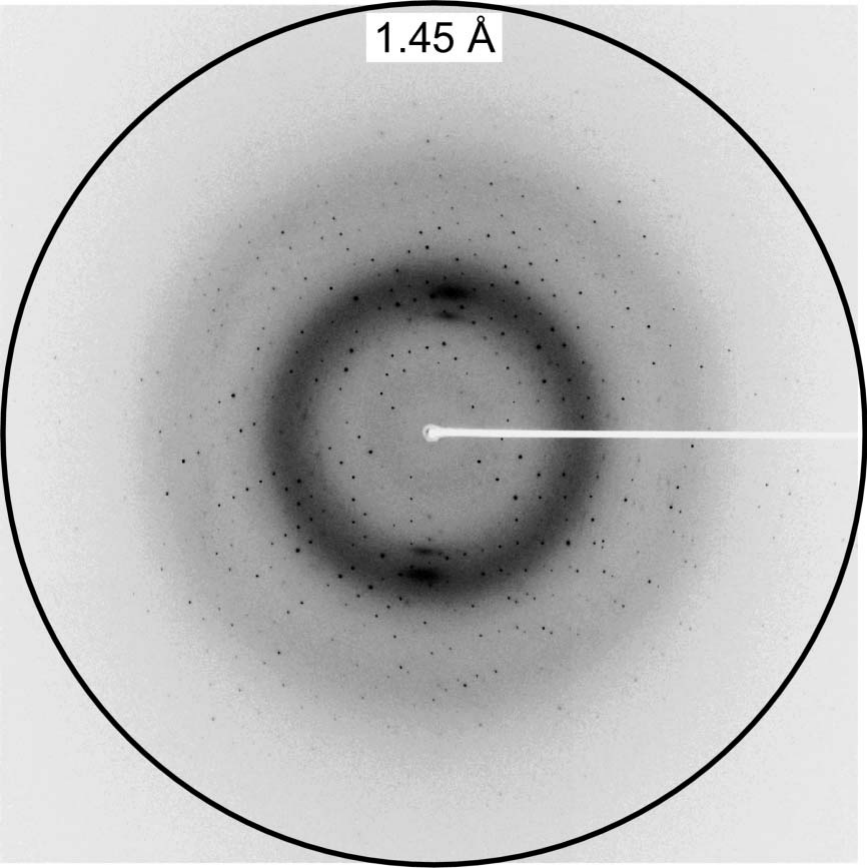
Diffraction image of the native crystal of YfcM. The circle indicates 1.45 Å resolution.

The crystal was used for the X-ray diffraction experiment. It diffracted X-rays to 1.45 Å resolution (Fig. 2 ▶) and belonged to space group *C*2, with unit-cell parameters *a* = 124.4, *b* = 37.0, *c* = 37.6 Å, β = 101.2°. The Matthews coefficient (*V*_M_) of the crystal was 1.91 Å^3^ Da^−1^, suggesting that one YfcM molecule is contained in one asymmetric unit, with a solvent content of 35.7% (Matthews, 1968 ▶). We are preparing selenomethionine-labelled YfcM crystals for structure determination.

## Figures and Tables

**Table 1:** Macromolecule-production information

Source organism	*E. coli*
DNA source	RIKEN BRC (catalogue No. JGD07547)
Forward primer†	GGGGGGGCATATGAACAGTACACACCACTA
Reverse primer‡	CCCCCTCGAGCGAGAGCGCTTCCGGCCACG
Cloning vector	pET-26b
Expression vector	pET-26b
Expression host	*E. coli* strain Rosetta2(DE3)
Complete amino-acid sequence of the construct produced§	MNSTHHYEQLIEIFNSCFADEFNTRLIKGDDEPIYLPADAEVPYNRIVFAHGFYASAIHEISHWCIAGKARRELVDFGYWYCPDGRDAQTQSQFEDVEVKPQALDWLFCVAAGYPFNVSCDNLEGDFEPDRVVFQRRVHAQVMDYLANGIPERPARFIKALQNYYYTPELTAEQFPWPEALSLEHHHHHH

† The *Nde*I site is underlined.

‡ The *Xho*I site is underlined.

§ The cloning artifact is underlined.

**Table 2:** Crystallization

Method	Hanging-drop vapour diffusion
Plate type	VIOLAMO plate
Temperature (K)	293
Protein concentration (mg ml^−1^)	7.9
Buffer composition of protein solution	20 m*M* Tris–HCl pH 8.0, 200 m*M* NaCl, 1 m*M* β-mercaptoethanol
Composition of reservoir solution	26% PEG 3350, 180 m*M* ammonium sulfate, 100 m*M* Bis-Tris pH 5.5
Volume and ratio of drop	4 µl, 1:1
Volume of reservoir (µl)	500

**Table 3:** Data collection and processing Values in parentheses are for the outer shell.

Diffraction source	SPring-8 BL32XU
Wavelength (Å)	1.0
Temperature (K)	100
Detector	MX225HE
Crystal-to-detector distance (mm)	133
Rotation range per image (°)	1.0
Total rotation range (°)	360
Exposure time per image (s)	1.0
Space group	*C*2
Unit-cell parameters (Å, °)	*a* = 124.4, *b* = 37.0, *c* = 37.6, α = γ = 90, β = 101.2
Mosaicity (°)	0.575
Resolution range (Å)	50–1.45 (1.48–1.45)
Total No. of reflections	148468
No. of unique reflections	28846 (1229)
Completeness (%)	95.8 (83.2)
Multiplicity	5.1 (2.6)
〈*I*/σ(*I*)〉	42.8 (2.8)
*R*_meas_	0.060 (0.307)
Overall *B* factor from Wilson plot (Å^2^)	24.6
